# Ethnic disparities, clinical and pathways to care characteristics associated with the offer, uptake, and type of psychological therapy during first-episode psychosis: examining the role of early intervention for psychosis

**DOI:** 10.1017/S0033291725101529

**Published:** 2025-09-05

**Authors:** Sherifat Oduola, Samir Pathan, Jo Hodgekins, Bonnie Teague, Thomas K.J. Craig, Robbin Murray, Craig Morgan

**Affiliations:** 1 School of Health Sciences, University of East Anglia, Norwich, UK; 2 Biomedical Research Centre, South London & Maudsley NHS Foundation Trust, London, UK; 3 Norwich Medical School, University of East Anglia, Norwich, UK; 4 Department of Psychosis Studies, Institute of Psychiatry, Psychology & Neuroscience, King’s College London, London, UK; 5 Department of Health Service and Population Research, Institute of Psychiatry, Psychology & Neuroscience, King’s College London, London, UK; 6 NSFT Research, Norfolk and Suffolk NHS Foundation Trust, Hellesdon Hospital, Norwich, UK

**Keywords:** CBT, early intervention, ethnicity, ethnic minority, first-episode psychosis, group therapy, psychological therapy, pathway to care, psychosis

## Abstract

**Background:**

Psychological therapy (PT) along with antipsychotic medication is the recommended first line of treatment for first-episode psychosis (FEP). We investigated whether ethnicity, clinical, pathways to care (PtC) characteristics, and access to early intervention service (EIS) influenced the offer, uptake, and type of PT in an FEP sample.

**Methods:**

We used data from the Clinical Record Interactive Search-First Episode Psychosis study. Inferential statistics determined associations between ethnicity, clinical, PtC, and PT offer/uptake. Multivariable logistic regression estimated the odds of being offered a PT and type of PT by ethnicity, clinical and PtC characteristics adjusting for confounders.

**Results:**

Of the 558 patients included, 195 (34.6%) were offered a PT, and 193 accepted. Cognitive behavioral therapy (CBT) (*n* = 165 of 195; 84.1%) was commonly offered than group therapy (*n* = 30 of 195; 13.3%). Patients who presented via an EIS (adj. OR = 2.24; 95%CI 1.39–3.59) were more likely to be offered a PT compared with those in non-EIS. Among the patients eligible for an EIS, Black African (adj. OR = 0.49; 95%CI = 0.25–0.94), Black Caribbean (adj. OR = 0.45; 95%CI = 0.21–0.97) patients were less likely to be offered CBT compared with their White British counterparts. Patients with a moderate onset of psychosis (adj. OR = 0.34; 95%CI = 0.15–0.73) had a reduced likelihood of receiving CBT compared with an acute onset.

**Conclusions:**

Accessing EIS during FEP increased the likelihood of being offered a PT. However, treatment inequalities remain by ethnicity and clinical characteristics.

## Introduction

Evidence has accumulated about ethnic inequalities in access to care and treatment for psychotic disorders. Compared with their White ethnic counterparts, people from Black and minority ethnic backgrounds are more likely to experience coercive treatment (Manuel et al., 2023; Morgan et al., 2005; Oduola et al., 2019), more likely to receive long-acting antipsychotic medications (Das-Munshi, Bhugra, & Crawford, 2018; Williams, Harowitz, Glover, Tek, & Srihari, 2020), more likely to be placed on community treatment orders (Patel et al., 2011), and less likely to receive psychological therapies (Colling et al., 2017; Schlief et al., 2023). Whilst antipsychotic medications are the mainstay treatment for psychotic disorders due to their efficacy in symptom reduction and treatment maintenance (Pacchiarotti et al., 2019), research has shown that augmenting pharmacological interventions with psychological and psychosocial interventions brings greater benefits to patients, including improved quality of life (Fusar-Poli et al., 2015), increased therapeutic alliance (Bhui et al., 2015), improved social functioning (Morrison et al., 2018), and better clinical outcomes (Morrison et al., 2020).

Current clinical guidance generally highlights the importance of offering psychological and psychosocial interventions for treating psychosis. In the UK, the National Institute for Clinical Excellence (NICE) recommends that people with first-episode psychosis should be offered psychological therapy (PT), such as cognitive behavioral therapy for psychosis (CBTp) and interventions involving the family (i.e. family intervention), along with antipsychotic medication (NICE, 2015). In the past decade, there has been a surge of interest in examining ethnic inequalities in receipt of psychological interventions among people with psychotic disorders. Evidence from the UK highlights pervasive inequalities in access to psychological therapy among ethnic minority people living with psychotic disorders. When surveying the National Clinical Audit of Psychosis data, Schlief et al. (2023) found that compared with White British people, every minoritized ethnic group, except those of mixed Asian-White and mixed Black African-White ethnicities, had lower adjusted odds of receiving CBTp. They also reported that people of Black African, Black Caribbean, non-African/Caribbean Black, non-British/Irish White, and of ‘any other’ ethnicity, also experienced lower adjusted odds of receiving family interventions (Schlief et al., 2023). Colling et al. (2017), in a sample of 2,308 patients with a diagnosis of schizophrenia disorders drawn from the electronic health records of a large mental health provider, showed that younger patients and white British patients were more likely to receive CBTp compared with people of Black ethnic groups (Colling et al., 2017).

These findings are echoed in the US. Oluwoye et al. (2018) employed data from the RAISE early treatment program to examine racial and ethnic differences in treatment outcomes among participants in a randomized controlled trial of an intervention for first-episode psychosis called NAVIGATE. They found that families of Hispanic participants in usual community care were less likely than non-Hispanic white families to receive family psychoeducation (Oluwoye et al., 2018). Similarly, Heun-Johnson et al. (2021) found in a sample of 3,017 privately insured patients that Black and Hispanic patients were less likely than White patients to receive psychotherapy from a behavioral health professional at FEP (Heun-Johnson et al., 2021).

Despite the growing evidence of ethnic inequalities in the offer of psychological therapies for psychosis, several methodological and clinical shortcomings need to be addressed. For example, a few of the previous studies have focused on populations of people with chronic forms of psychotic illness (Colling et al., 2017; Das-Munshi et al., 2018; Mercer, Evans, Turton, & Beck, 2019) and are consequently, biased toward those likely to have poor engagement with services. Others have used early intervention for psychosis as a proxy for first-episode psychosis (Schlief et al., 2023), so it remains unclear whether and how the NICE-recommended treatment is being delivered to people with FEP during a first presentation to mental health services. In addition, most of the literature has been ‘silent’ regarding the influence of pathways to care and clinical characteristics during FEP on the offer of psychological therapy. For example, duration of untreated psychosis (DUP), usually defined as the time from the onset of frank psychotic symptoms (i.e. hallucinations or delusions) to the date of first contact with a mental health service for psychosis or the start of antipsychotic treatment (Singh, 2007), and the speed at which psychotic symptoms develop (i.e. mode of onset) (Compton, Chien, Leiner, Goulding, & Weiss, 2008) are important indicators of illness prognosis. Indeed, a sizeable body of evidence has shown that a prolonged DUP is associated with poor clinical outcomes (Drake, Haley, Akhtar, & Lewis, 2000; Marshall et al., 2005), reduced social functioning, and poor quality of life (Craig et al., 2000; Marshall et al., 2005). However, the extent to which DUP or mode of onset influences access to psychological therapy is unclear. To address these gaps, in this study, we used an epidemiologically derived cohort of first-episode psychosis patients to examine whether (a) ethnicity, pathways to care, and clinical characteristics influenced the offer of psychological therapies in an FEP sample, (b) there were ethnic and clinical differences in the offer, uptake, and type of psychological therapies, and (c) early intervention for psychosis service played a role in the offer and type of psychological therapies.

## Methods

### Settings, study design, data source, and participants

This study was carried out in the inner-city London boroughs of Lambeth and Southwark, served by the South London and Maudsley NHS Trust (SLaM). SLaM provides mental healthcare for the residents of five boroughs in south-east London, with a total population of 1.3 million (Perera et al., 2016). Adult services for patients with psychotic disorders in SLaM comprise community, outpatient, and inpatient teams.

Participants in this study were drawn from a large incidence study, namely the Clinical Record Interactive Search—First Episode Psychosis (CRIS-FEP) study (Oduola et al., 2021b). Patients presenting for the first time with FEP (i.e. ICD F20–29, F30–33) to any adult mental health service in SLaM between May 2010 and April 2012 were identified. Data were obtained from the SLaM Biomedical Research Centre Clinical Records Interactive Search (CRIS) system (Perera et al., 2016; Stewart et al., 2009), which provides fully de-identified access to all SLaM electronic clinical records. CRIS is a bespoke research database search and assembly tool which has supported several studies (Colling et al., 2017; Das-Munshi et al., 2017; Patel et al., 2017). The clinical information documented in CRIS is available in structured fields (for diagnosis and demographic information) and unstructured free-text fields (for clinical notes and correspondence).

### Procedure

#### Case identification

The approach for identifying cases in the CRIS-FEP study has been described and published previously (Oduola, Craig, Iacoponi, Macdonald, & Morgan, 2023; Oduola, Das-Munshi, et al., 2021b). In summary, the Structured Query Language (SQL) (SQL – ANSI, 2011) was used to interrogate the structured and free-text fields in CRIS to retrieve the records of patients presenting to any adult mental health services between 2010 and 2012; then we applied defined search terms (e.g. ‘psychos*’; ‘onset’; ‘psychosis’; ‘voices’). This returned records of probable participants. Second, the research team screened each patient’s de-identified records for eligibility using the Screening Schedule for Psychosis (Jablensky et al., 1992) and the study inclusion/exclusion criteria.

#### Inclusion/exclusion criteria

Participants were included if they were residents in the London boroughs of Lambeth or Southwark, (b) aged 18–64 years old (inclusive) at presentation, (c) with a clinical diagnosis of a psychotic disorder (i.e. ICD F20–29, F30–33), and (d) were in first contact with mental health services for psychosis. Exclusion criteria were (a) evidence of psychotic symptoms with an organic cause, (b) transient psychotic symptoms resulting from acute intoxication, and (c) previous contact with services for psychotic symptoms.

#### Early intervention for psychosis and eligibility

Early intervention for psychosis services (EIS) are designed to identify and provide appropriate interventions as early as possible during an individual’s first episode of psychosis to reduce treatment delays and improve outcomes (Singh, 2010). At the time of this study, FEP patients accessed mental health services at SLaM by two routes EIS and non-EIS depending on eligibility. The main eligibility criterion for accessing an EIS in SLaM at the time of our study was age, i.e. 18–35 years. This was before the introduction of the Access and Waiting Time Standard, i.e. April 1, 2016, when the upper age limit was extended to 65 years in England (NHS England, 2016). Therefore, our analyses relating to the role of EIS in the offer or type of PT were restricted to those aged 18–35 years. Early intervention psychosis services at SLaM typically offer a 3-year duration of treatment and support (Oduola et al., 2023).

#### Demographic characteristics

The procedure for extracting sociodemographic and clinical data for the CRIS-FEP sample has been reported elsewhere (Oduola et al., 2019; Oduola, Craig, & Morgan, 2021a). In summary, the Medical Research Council Socio-demographic schedule MRC-SDS (Mallett, 1997) was used to capture data on demographic variables. Ethnicity was coded according to the 18 categories in the UK 2011 census (ONS, 2011). We collapsed the ethnic groups into seven categories for the purpose of analysis as follows: White British, Black Caribbean (Black Caribbean and Other Black), Black African, Asian (Indian, Pakistani, Bangladeshi, Chinese), White non-British (White Irish, White Gypsy, White Other), Other (Arab, Any Other Ethnic group), and Mixed (all Mixed ethnic groups). The ethnic characteristics of the sample in this study are representative of the study catchment areas, although there is heterogeneity when compared with England, as shown in Supplementary Table S1.

#### Clinical variables

Data relating to clinical and pathways to care characteristics, including duration of untreated psychosis (DUP), mode of onset of psychosis, and access to EIS, were collected using the Personal and Psychiatric History Schedule (PPHS) (WHO, 1996). Mode of onset of psychosis is defined as the speed at which psychotic symptoms develop, including an acute onset (within days or a week) or in a more gradual way, for more than a few months (Compton et al., 2008). This information was captured as a categorical variable in the PPHS. DUP was measured as a continuous variable (in days) from the date of onset of psychotic symptoms as recorded in clinical records to the date of first contact with SLaM for first-episode psychosis.

#### Outcome variables

The outcome data were the offer, uptake, and type of psychological therapy. Psychological therapy was defined as any formal therapy or psychosocial intervention, including CBTp, family intervention, group therapy, or counselling. Outcome data were collected from the study’s inception, from May 2010 until April 2014. Data on offer, acceptance, and type of psychological therapy were manually extracted from the electronic health records’ structured and free-text fields, using an adapted version of the Life Chart Schedule for case notes (Harrison et al., 2001). We operationalized the adapted Life Chart Schedule for data extraction from CRIS by first retrieving the clinical records of each patient in the CRIS-FEP study sample. Second, we interrogated the free-text fields of CRIS (including clinical assessment, correspondence, and discharge summaries) to determine whether the patient was offered and accepted a psychological therapy, both coded as binary variables: ‘yes’ or ‘no’ and the type of psychological therapy offered. Two researchers who have been trained in clinical records data extraction conducted the data collection. Inter-rater reliability was done on 20% of the sample on the offer of PT and type of PT variables between the two raters. Kappa scores of k = 0.97, p < 0.001 and k = 0.83, p < 0.001 were achieved, respectively between the raters, indicating a substantial agreement. Discrepant or ambiguous cases were resolved by consensus with research team members.

#### Statistical analysis

Stata version 15 was used to analyze the data (StataCorp, 2017). Descriptive statistics for the outcome and exposure variables were obtained as frequencies and percentages for categorical variables and mean (standard deviation [SD]) and median (interquartile range [IQR]) for continuous variables. We performed chi-square and Kruskal Wallis tests (as appropriate) to compare demographic and clinical characteristics between patients offered and not-offered PT. We performed univariable and multivariable logistic regression analyses to (a) examine associations between ethnicity, clinical, PtC factors, and offer of a PT in the whole sample; (b) assess associations between ethnicity, clinical factors, and offer of PT in the sample of patients aged 18–35 years who were eligible for an EIS; and (c) examine associations between the type of PT offered (CBTp versus no-CBTp), clinical characteristics, and ethnicity in those eligible for EIS. In all the multivariable regression models, we adjusted for *a-prior* confounders (age and gender) and other variables in the models. There were two missing data in the EIS variable, and those were removed from the analyses. We performed Bonferroni confidence interval adjustments (Curtin & Schulz, 1998) for multiple comparisons when relevant.

## Ethical approval

The Oxfordshire Research Ethics Committee approved the CRIS system as an anonymized dataset for secondary analysis (reference 23/SC/0257). We obtained local approval for this study via the CRIS Oversight Committee at the BRC South London and Maudsley NHS Foundation Trust (reference: 09–041). Under UK law, patient consent was not required for this study.

## Results

### Sample characteristics

Table 1 summarizes the patients’ characteristics. Five hundred and fifty-eight FEP patients were identified. The mean age was 33.6 (SD:10.6) years; there were more men (52.3%) and Black African people (26.3%) than other ethnic groups. Most of the patients did not access an EIS (58.1%), an insidious onset of psychosis is common (37.5%), and a median DUP of 93 (IQR: 19–447) days was observed.

**Table 1.** Sample characteristics

	N = 558	%
**Mean age (SD) years.**	33.62 (10.6)	
**Sex**		
Male	292	52.3
Female	266	47.7
**Ethnicity**		
White British	133	23.8
Black African	147	26.3
Black Caribbean	91	16.3
White other	75	13.4
Asian	44	7.9
Mixed	27	4.8
Other	41	7.4
**EIS eligibility**		
Yes (aged 18–35 years)	340	60.9
No (aged <35 years)	218	39.1
**Pathway to care^a^**		
EIS (received)	222	39.9
Other service	334	58.1
**Mode of Onset of FEP**		
Acute	116	20.7
Moderate	111	19.9
Gradual	122	21.9
Insidious	209	37.5
**Duration of untreated psychosis** (DUP; IQR), days	93	19–447
Offer of psychological therapy		
No	362	65.4
Yes	195	34.6
**Type of psychological therapy**		
CBT	165	86.4
Group therapy	30	13.6
**Accepted offer**		
Yes	193	98.4
No	2	1.6

a 2 missing data

### Offer, uptake, and type of psychological therapy

One hundred and ninety-five people were offered psychological therapy, of whom only two (1.6%) declined. Two types of PT were offered, namely CBTp (86.4%) and group therapy (13.6%) (Table 1).

### Associations between demographic, pathways to care characteristics, and offer of any psychological therapy

We found that characteristics of the pathways to care and mode of onset of psychosis were associated with the offer of psychological therapy. Patients in the EIS were more likely to be offered a PT compared to those in the non-EIS group (EIS: 44.8% vs non-EIS 28.5%, p < 0.0001). This was reflected in the differences observed by age, in which those aged 18–35 years (37.6%) were more likely to be offered a PT than those aged 36–64 (30.6%; p = 0.08). We found that patients with an acute onset of psychosis were more likely to be offered a PT (PT offered: 44.0%) compared with a moderate onset (PT offered: 27.3%). There were no clear differences in the offer of PT by DUP or sex (Table 2).

**Table 2.** Demographic, clinical, pathways to care characteristics by offer of any psychological therapy (PT)

	Offer of any PT			
Variables	No *n* = 362	Yes *n* = 195	*X*^2^, Kruskal Wallis tests (df)	*p*
**Age-band**			**2.92 (1)**	0.08
**18–35 years**	212 (62.4)	128 (37.6)		
**36–64 years**	150 (69.4)	66 (30.6)		
**Sex, *n* (%)**				
Male	193 (66.3)	98 (33.7)		
Female	169 (63.8)	96 (36.2)		
**Ethnicity, *n* (%)**			9.65 (6)	0.14
White British	76 (57.1)	57 (42.9)		
Black African	95 (65.1)	51 (34.9)		
Black Caribbean	60 (65.9)	31 (34.1)		
White non-British	54 (72.0)	21 (28.0)		
Asian	31 (70.5)	13 (29.5)		
Mixed	15 (55.6)	12 (44.4)		
Other	31 (77.5)	9 (22.5)		
**Pathways to care**			**16.06 (1)**	**<0.001**
Non-Early Intervention Service (EIS)	238 (71.5)	94 (28.5)		
Early Intervention Service	122 (55.2)	99 (44.8)		
**DUP, median (IQR)**	94 (21–512)	93 (11–361)	1.64 (1)	0.19
**Mode of onset, *n* (%)**			**8.85 (3)**	**0.03**
Acute	65 (56.0)	51 (44.0)		
Moderate	80 (72.7)	30 (27.3)		
Gradual	74 (61.2)	47 (38.8)		
Insidious	143 (68.4)	66 (31.6)		

### Associations between the offer of any psychological therapy and ethnic, clinical, and PtC characteristics

In the whole sample (*n* = 556), we found strong evidence that patients accessing EIS were twice as likely to be offered a PT (adj. OR: 2.24 [95%CI:1.39–3.59]) compared with non-EIS patients. We also found that patients with a moderate mode of onset of psychosis were less likely to be offered any PT (adj. OR: 0.52 [95%CI: 0.29–0.92]) compared with those with an acute onset. There was insufficient evidence of ethnic differences in the offer of any PT (Table 3).

**Table 3.** Unadjusted and adjusted odds ratios of associations between ethnicity, clinical, pathways to care and offer of any psychological therapy in the full sample (*n* = 556)

Variables	Model 1 (95% CI)	Model 2 (95% CI)	Bonferroni corrected 95% CI
**Ethnicity**			
White British	1.00	1.00	
Black African	0.71 (0.44–1.16)	0.67 (0.40–1.10)	−1.16 – 0.37
Black Caribbean	0.68 (0.39–1.19)	0.68 (0.38–1.21)	−1.26 – 0.50
White non-British	**0.51 (0.28–0.95)**	0.54 (0.29–1.01)	−1.56 – 0.36
Asian	0.55 (0.26–1.16)	0.57 (0.26–1.23)	−1.73 – 0.63
Mixed	1.06 (0.46–2.45)	1.09 (0.46–2.58)	−1.24 – 1.42
Other	**0.38 (0.17–0.87)**	0.38 (0.18–1.01)	−2.13 – 0.48
**Pathways to care**			
Non-EIS	**1.00**	**1.00**	*n*/a
EIS	**2.03 (1.42–2.90)**	**2.24 (1.39–3.59)**	
DUP	0.99 (0.99–1.00)	0.99 (0.99–1.00)	*n*/a
**Mode of onset**			
Acute	1.00	**1.00**	
Moderate	**0.47 (0.27–0.83)**	**0.52 (0.29–0.92)**	−1.42 – −0.12
Gradual	0.80 (0.48–1.35)	0.82 (0.48–1.40)	−0.91 – 0.52
Insidious	**0.58 (0.36–0.93)**	0.63 (0.37–1.07)	−1.17 – 0.25
**Age**	0.98 (0.97–1.00)	1.01 (0.99–1.03)	*n*/a
**Female**	1.11 (0.78–1.58)	1.18 (0.81–1.71)	*n*/a

CI confidence interval

Model 1 unadjusted

Model 2 age, gender, ethnicity, DUP, mode of onset, EIS.

When we assessed ethnic differences in the offer of PT among patients who were eligible for an EIS, there was no evidence of associations. However, patients with a moderate onset of psychosis remained less likely to be offered any PT (adj. OR: 0.40 [95%CI: 0.19–0.82]) compared with those with an acute onset (Table 4). These results were held after Bonferroni corrections.

**Table 4.** Unadjusted and adjusted odds ratios of associations between ethnicity, clinical and offer of any psychological therapy (*n* = 340) in patients eligible for EIS

Variables	Model 1 (95% CI)	Model 2 (95% CI)	Bonferroni corrected 95% CI
**Ethnicity**			
White British	1.00	1.00	
Black African	0.71 (0.38–1.31)	0.69 (0.37–1.29)	−1.33 – 0.60
Black Caribbean	0.72 (0.35–1.48)	0.66 (0.32–1.38)	−1.53 – 0.73
White non-British	0.53 (0.23–1.20)	0.50 (0.21–1.16)	−1.97 – 0.61
Asian	0.51 (0.20–1.26)	0.47 (0.18–1.19)	−2.18 – 0.69
Mixed	1.20 (0.44–3.21)	1.13 (0.41–3.10)	−1.43 – 1.63
Other	0.42 (0.15–1.10)	0.44 (0.16–1.21)	−2.34 – 0.73
**DUP**	0.99 (0.99–1.00)	0.99 (0.99–1.00)	*n*/a
**Mode of onset**			
Acute	1.00	1.00	
Moderate	**0.42 (0.20–0.86)**	**0.40 (0.19–0.82)**	**−**1.89 – −0.06
Gradual	1.09 (0.58–2.04)	1.04 (0.55–1.98)	−0.81 – 0.90
Insidious	**0.54 (0.30–0.96)**	0.57 (0.29–1.12)	−1.43 – 0.34
**Age**	0.97 (0.93–1.02)	0.99 (0.94–1.04)	*n*/a
**Female**	0.90 (0.99–1.42)	0.88 (0.55–1.41)	*n*/a

CI confidence interval

Model 1 unadjusted

Model 2 adjusted for age, gender, ethnicity, DUP, mode of onset.

### Ethnic and clinical differences in the type of psychological therapy offered (i.e. CBTp versus non-CBTp) among patients eligible for early intervention services

In Table 5, we assessed ethnic variations in the type of PT offered to patients eligible for EIS. We focused on CBTp given it was the type of PT mostly offered. Multivariable logistic regression analyses showed strong evidence that Black African (adj. OR: 0.49 [95% CI: 0.25–0.94]) and Black Caribbean (adj. OR: 0.45 [95% CI: 0.21–0.97]) patients were less likely to be offered CBTp (relative to non-CBTp) compared with their White British counterparts. A moderate onset of psychosis was also associated with a reduced odds of being offered CBTp (adj. OR: 0.34 [95%CI: 0.15–0.73]). These results were held after Bonferroni corrections. We found no evidence of associations between the offer of CBT and DUP.

**Table 5.** Unadjusted and adjusted odds ratios of associations between ethnicity, clinical characteristics and offer of CBT in patients eligible for EIS (*n* = 340)

Variable	Model 1 (95% CI)	Model 2 (95% CI)	Bonferroni corrected 95% CI
**Ethnicity**			
White British	1.00	1.00	
Black African	**0.50 (0.27–0.96)**	**0.49 (0.25–0.94)**	−1.71 – – 0.29
Black Caribbean	**0.49 (0.23–1.05)**	**0.45 (0.21–0.97)**	−1.97 – −0.40
White non-British	0.56 (0.24–1.27)	0.54 (0.23–1.25)	−1.90 – 0.68
Asian	0.45 (0.18–1.16)	0.41 (0.15–1.07)	−2.36 – 0.59
Mixed	1.26 (0.47–3.38)	1.22 (0.44–3.35)	−1.36 – 1.74
Other	**0.36 (0.13–0.99)**	0.36 (0.13–1.08)	−2.55 – 0.65
**DUP**	0.99 (0.99–1.00)	0.99 (0.99–1.03)	*n*/a
**Mode of onset**			
Acute	1.00	1.00	
Moderate	**0.37 (0.17–0.78)**	**0.34 (0.15–0.73)**	−2.11 – –0.40
Gradual	0.98 (0.48–1.72)	0.86 (0.44–1.65)	−1.02 – 0.73
Insidious	**0.53 (0.29–0.96)**	0.57 (0.28–1.13)	−1.47 – 0.36
**Age**	0.98 (0.97–1.00)	0.98 (0.94–1.03)	*n*/a
**Female**	1.11 (0.78–1.58)	0.97 (0.60–1.58)	*n*/a

CI: confidence interval

Model 1: unadjusted

Model 2: adjusted for age, gender, ethnicity, DUP, mode of onset.

## Discussion

### Main findings

In this study, we investigated ethnic, PtC and clinical disparities in the offer, uptake, and type of psychological therapy during the first episode of psychosis. We assessed demographic, clinical, and pathways to care factors associated with being offered a psychological therapy and the type offered. Our sample is representative of people with psychotic illness who access and receive care from inner-city mental health services in the UK. We found that most patients offered a PT were those receiving care from an EIS, which is corroborated by the finding that younger patients aged 18–35 years were more likely to be offered a PT. It is noteworthy that EIS typically accepted patients aged 18–35 years at the time of our study. All but *n* = 2 of 195; (1.16%) patients accepted the offer of PT, suggesting there is a willingness to accept treatment. Patients were offered either CBTp or group therapy, with the majority being offered CBTp. Overall, we found insufficient evidence of ethnic differences in the offer of any psychological therapy. However, we found strong evidence of association between mode of onset of psychosis, indicating that patients with a moderate onset were less likely to be offered a PT. Initially, the multivariable analyses of patients eligible for an EIS (i.e. aged 18–35 years old) suggested there was no ethnic variation in the offer of PT among those eligible for EIS. However, when we assessed the associations by type of PT offered (i.e. CBTp versus non-CBTp), our analyses indicated large variations by ethnicity and clinical factors. We found that compared with White British patients, Black African and Black Caribbean patients were less likely to be offered CBTp. Patients with a moderate onset of psychosis were also less likely to be offered CBTp.

### Methodological considerations

The findings need to be considered in the context of some methodological limitations. First, data were drawn from clinical records; therefore, reporting accuracy depends on the quality of clinicians’ documentation. Although clinicians are required to document treatment offered to patients, some patients may have been offered PT but not documented in the records. Second, our data were collected before the UK government introduced the Access and Waiting Time Standards (AWTS) (NHS England, 2016); despite this, our findings are comparable to more recent studies. Third, while we did not measure or adjust for socioeconomic factors when examining ethnic differences in the offer and type of PT, several lines of reasoning suggest this is unlikely to bias our findings. For instance, Schlief et al. (2023) found lower odds of being offered CBTp among all minority ethnic groups after controlling for socioeconomic variables. Fourth, our findings in some ethnic groups, e.g. the ‘other’ and white non-British patients may not be generalizable owing to the heterogeneity of these ethnic groups. We included people of white Irish, white Gypsy/traveler, white non-British ethnicities in our white non-British, and people identifying as Arab and any other ethnic group were included in our ‘other’ ethnic group. Whilst we adjusted for several sociodemographic, PtC, and clinical factors, unmeasured factors, such as socioeconomic status, clinician bias, cultural stigma, systemic barriers, or cultural perceptions of therapy, may still confound the results.

Despite these limitations, our study has several methodological strengths. We used an epidemiologically derived cohort of people with first-episode psychosis assembled within the CRIS-FEP study (Oduola et al., 2023; Oduola, Das-Munshi, et al., 2021b), providing a basis to determine the treatment trajectory at the start of the illness. We comprehensively reviewed the de-identified electronic health records of every CRIS-FEP patient for up to approximately 4 years to carefully determine their offer, type and uptake of PT. There were only two patients with missing data in our analysis in determining ethnic and clinical disparities in offer and type of PT, hence minimizing bias. Another strength is that our large sample size allowed us to categorize ethnicity according to the UK Census Ethnic Classifications, and the ethnic characteristics of our sample are representative of the base population (ONS, 2011). Furthermore, to our knowledge, this is one of the few studies that have considered the influence of pathways to care and clinical characteristics on the offer of PT during first-episode psychosis.

### Interpretations of findings and relationship to previous studies

Our findings of ethnic variations in the type of PT (i.e. CBTp) align with many previous studies (Colling et al., 2017; Das-Munshi et al., 2018; Mercer et al., 2019; Oluwoye et al., 2018; Schlief et al., 2023). Specifically, the low offer of CBTp to the Black African (adj. OR:0.49 [95% CI: 0.25–0.94]) and Black Caribbean (adj. OR: 0.45 [95% CI: 0.21–0.97]) patients in our study has been shown in recent studies. For example, Schlief et al. (2023) found adj. OR: 0.53 (95% CI: 0.47–0.59) and adj. OR: 0.59 (95% CI: 0.51–0.69) for CBTp among Black African and Black Caribbean patients, respectively. These findings were also echoed by Colling et al. (2017). However, contrary to previous studies (Mercer et al., 2019; Schlief et al., 2023), we did not find sufficient evidence of a reduced likelihood of the offer of any PT by ethnicity.

Considering pathways to care, DUP, and mode of onset of psychosis, we found that a high proportion of patients accessing early intervention services were offered a PT, and the majority accepted the treatment. This is not surprising, as a key treatment approach for FEP within early intervention for psychosis services is psychological therapy, according to the NICE guidelines (NICE, 2015). Our observation is also consistent with the National Clinical Audit of Psychosis (Royal College of Psychiatrists, 2022), which examined the rates of offer and receipt of therapy in early intervention for psychosis teams in England and found that an average of 86% of service users with psychosis were offered CBTp in 2021/22. However, the uptake of psychological therapy was greater in our study (98.4%) compared with 46% of service users taking up the offer of CBTp in the National Clinical Audit of Psychosis audit (Royal College of Psychiatrists, 2022).

We did not find evidence of associations between DUP and the offer or type of PT. However, we observed that an acute onset of psychosis is common among patients who were offered a PT, and conversely, those with a moderate onset were less likely to be offered any PT and more specifically CBTp. This observation may be explained by considering symptom recognition, i.e. frank psychotic symptoms are more recognizable in an acute presentation than in a moderate or more gradual onset of psychosis. Indeed, significant efforts have been spent in reducing treatment delays and improving outcomes of psychosis, and part of achieving this lies in recognizing the symptoms and initiating help-seeking. A recent systematic review of public health interventions, campaigns, and initiatives designed to improve pathways to care for individuals with psychotic disorders shows that interventions targeting multiple populations (general public and non-healthcare professionals) and those lasting >12 months show promise for reducing the duration of untreated psychosis (Murden, Allan, Hodgekins, & Oduola, 2024). Therefore, we could argue that if patients are able to access specialist psychosis services quicker for treatment, i.e. shorter DUP, then they are more likely to access psychological therapy.

Furthermore, our data suggest that younger patients were more likely to be offered a PT in keeping with previous findings (Colling et al., 2017; Heun-Johnson et al., 2021; Schlief et al., 2023). This may be linked to the age of onset of psychosis, which tends to occur in late teens and early adulthood (Oduola, Das-Munshi, et al., 2021b). Additionally, we collected data for this study when the age of acceptance to early intervention for psychosis services was between 18 and 35 years; therefore, it is logical that younger people accessed EIS, which meant they were more likely to be offered a PT. Future study assessing variations in psychological therapy by age would be helpful. This is particularly important in the UK, given the implementation of the Access and Waiting Time Standards (NHS England, 2016), which recommends that EIS accept patients up to 65 years of age. Therefore, further research using post-AWTS data and the inclusion of older patients may provide insights into current practices and the uptake of PT. In contrast to previous studies (Das-Munshi et al., 2018; Schlief et al., 2023), we found no patient was offered a family intervention. This is interesting, given the NICE guidelines and research demonstrating the benefits of family intervention in FEP populations (Claxton, Onwumere, & Fornells-Ambrojo, 2017).

### Implications for clinical practice

Implications for clinical practice are highlighted in our results. We found significant under-presentation of Black African and Black Caribbean patients being offered CBTp. One possible explanation could be linked to the mismatch in the demographic characteristics of the patient population and the clinicians who deliver psychological therapies. It is well documented in clinical psychology that there is a lack of representation of Black and minority ethnic group people in the workforce (Turpin & Coleman, 2010; Wood & Patel, 2017). However, there is good evidence that in the dyad of patient-therapist matched on race/ethnicity, significant improvement in functioning was observed over time (Duong et al., 2024). Cooper and colleagues, in a qualitative study, reported that clinicians’ implicit racial bias was associated with Black patients’ perceptions of poorer communication and lower ratings of quality of care (Cooper et al., 2012). Another important consideration is the notion of cultural sensitivity, which is the extent to which services and healthcare professionals are sensitive to people’s cultural identity or heritage, including ethnicity (Care Quality Commission, 2024). Previous studies have shown when healthcare professionals are curious about patients’ cultural identity, beliefs, and heritage, patients feel heard, accepted, and supported (Conneely et al., 2023; Gardner 2024). There are calls for clinical psychologists to be more representative of the local population’s culture and personal identities that they serve (Mercer et al., 2019). In the UK, it is noteworthy that the Department of Health and Social Care aims to increase and improve the diversity of its workforce (NHS England, 2023). Nonetheless, it remains imperative that ethnic inequalities are explored and addressed to provide equitable healthcare for all.

### Future research

This study provides important findings about demographic, clinical, and pathways to care factors associated with the offer, uptake, and type of psychological therapies during FEP within a diverse urban population. Future research across diverse catchment areas is warranted to validate these findings. For example, it would be beneficial for future research to investigate differences in access to psychological therapies in rural populations and gender minority groups, e.g. LGBTQ+ people. Additionally, understanding the mechanisms underlying the ethnic inequalities in accessing NICE-recommended treatment is critical. This could be achieved through qualitative approaches involving patients, carers, and clinicians, and, more importantly, such research could be co-produced with people with lived experience.

## Conclusions

Our study shows that accessing an early intervention service during FEP increased the likelihood of being offered a PT. However, treatment inequalities remain by ethnicity and clinical characteristics. Our findings are relevant to international policymakers, clinicians, patients, and carers. Improving access to psychological therapies and targeting provision toward underserved groups are critical. Greater efforts are needed to ensure people at all stages of a psychotic illness receive treatment and interventions in an equitable manner, which in turn will improve outcomes.

## Supporting information

Oduola et al. supplementary material Oduola et al. supplementary material

## Data Availability

No additional data are available.

## Supplementary material

The supplementary material for this article can be found at http://doi.org/10.1017/S0033291725101529.

## References

[r1] Bhui, K. S., Aslam, R. W., Palinski, A., McCabe, R., Johnson, M. R., Weich, S., Singh, S. P., Knapp, M., Ardino, V., & Szczepura, A. (2015). Interventions to improve therapeutic communications between black and minority ethnic patients and professionals in psychiatric services: Systematic review. The British Journal of Psychiatry, 207, 95–103. 26243761 10.1192/bjp.bp.114.158899 PMC4523926

[r2] Care Quality Commission. (2024). Culturally appropriate care. Care Quality Commission.

[r3] Claxton, M., Onwumere, J., & Fornells-Ambrojo, M. (2017). Do family interventions improve outcomes in early psychosis? A systematic review and meta-analysis. Frontiers in Psychology, 8, 371. 28396643 10.3389/fpsyg.2017.00371 PMC5366348

[r4] Colling, C., Evans, L., Broadbent, M., Chandran, D., Craig, T. J., Kolliakou, A., Stewart, R., & Garety, P. A. (2017). Identification of the delivery of cognitive behavioural therapy for psychosis (CBTp) using a cross-sectional sample from electronic health records and open-text information in a large UK-based mental health case register. BMJ Open, 7, e015297. 10.1136/bmjopen-2016-015297 PMC5734297 28716789

[r5] Compton, M. T., Chien, V. H., Leiner, A. S., Goulding, S. M., & Weiss, P. S. (2008). Mode of onset of psychosis and family involvement in help-seeking as determinants of duration of untreated psychosis. Social Psychiatry and Psychiatric Epidemiology, 43, 975–982. 18604616 10.1007/s00127-008-0397-y

[r6] Conneely, M., Packer, K. C., Bicknell, S., Janković, J., Sihre, H. K., McCabe, R., Copello, A., Bains, K., Priebe, S., Spruce, A., & Jovanović, N. (2023). Exploring black and south Asian women’s experiences of help-seeking and engagement in perinatal mental health services in the UK. Frontiers in Psychiatry, 14, 1119998. 37077277 10.3389/fpsyt.2023.1119998 PMC10109459

[r7] Cooper, L. A., Roter, D. L., Carson, K. A., Beach, M. C., Sabin, J. A., Greenwald, A. G., & Inui, T. S. (2012). The associations of clinicians’ implicit attitudes about race with medical visit communication and patient ratings of interpersonal care. American Journal of Public Health, 102, 979–987. 22420787 10.2105/AJPH.2011.300558 PMC3483913

[r8] Craig, T. J., Bromet, E. J., Fennig, S., Tanenberg-Karant, M., Lavelle, J., & Galambos, N. (2000). Is there an association between duration of untreated psychosis and 24-month clinical outcome in a first-admission series? American Journal of Psychiatry, 157, 60–66. 10618014 10.1176/ajp.157.1.60

[r9] Curtin, F., & Schulz, P. (1998). Multiple correlations and bonferroni’s correction. Biological Psychiatry, 44, 775–777. 9798082 10.1016/s0006-3223(98)00043-2

[r10] Das-Munshi, J., Bhugra, D., & Crawford, M. J. (2018). Ethnic minority inequalities in access to treatments for schizophrenia and schizoaffective disorders: Findings from a nationally representative cross-sectional study. BMC Medicine, 16, 55. 29669549 10.1186/s12916-018-1035-5 PMC5904997

[r11] Das-Munshi, J., Chang, C. K., Dutta, R., Morgan, C., Nazroo, J., Stewart, R., & Prince, M. J. (2017). Ethnicity and excess mortality in severe mental illness: A cohort study. Lancet Psychiatry, 4, 389–399. 28330589 10.1016/S2215-0366(17)30097-4 PMC5406616

[r12] Drake, R. J., Haley, C. J., Akhtar, S., & Lewis, S. W. (2000). Causes and consequences of duration of untreated psychosis in schizophrenia. The British Journal of Psychiatry, 177, 511–515. 11102325 10.1192/bjp.177.6.511

[r13] Duong, L. A., Zoupou, E., Boga, C. I., Kashden, J., Fisher, J., Connolly Gibbons, M. B., & Crits-Christoph, P. (2024). Gender, race/ethnicity, and patient-therapist matching on gender and race/ethnicity: Predictors/moderators of the effectiveness of trust/respect feedback. Administration and Policy in Mental Health, 52, 59–73. 38175334 10.1007/s10488-023-01335-1 PMC11750298

[r14] Fusar-Poli, P., Frascarelli, M., Valmaggia, L., Byrne, M., Stahl, D., Rocchetti, M., Codjoe, L., Weinberg, L., Tognin, S., Xenaki, L., & McGuire, P. (2015). Antidepressant, antipsychotic and psychological interventions in subjects at high clinical risk for psychosis: OASIS 6-year naturalistic study. Psychological Medicine, 45, 1327–1339. 25335776 10.1017/S003329171400244X

[r50] Gardner, A., Oduola, S., & Teague, B. (2024). Culturally Sensitive Perinatal Mental Health Care: Experiences of Women From Minority Ethnic Groups. Health Expect, 27(4):e14160. doi: 10.1111/hex.14160. 39087742 PMC11292667

[r15] Harrison, G., Hopper, K., Craig, T., Laska, E., Siegel, C., Wanderling, J., Dube, K. C., Ganev, K., Giel, R., An der Heiden, W., Holmberg, S. K., Janca, A., Lee, P. W. H., León, C. A., Malhotra, S., Marsella, A. J., Nakane, Y., Sartorius, N., Shen, Y., … Wiersma, D. (2001). Recovery from psychotic illness:: A 15-and 25-year international follow-up study. British Journal of Psychiatry, 178, 506–517. 10.1192/bjp.178.6.506 11388966

[r16] Heun-Johnson, H., Menchine, M., Axeen, S., Lung, K., Claudius, I., Wright, T., & Seabury, S. A. (2021). Association between race/ethnicity and disparities in health care use before first-episode psychosis among privately insured young patients. JAMA Psychiatry, 78, 311–319. 33355626 10.1001/jamapsychiatry.2020.3995 PMC7758828

[r17] Jablensky, A., Sartorius, N., Ernberg, G., Anker, M., Korten, A., Cooper, J., Day, R., & Bertelsen, A. (1992). Schizophrenia: Manifestations, incidence and course in different cultures a World Health Organization ten-country study. Psychological Medicine. Monograph Supplement, 20, 1–97. 1565705 10.1017/s0264180100000904

[r18] Mallett, R. (1997). Sociodemographic schedule. London: Section of Social Psychiatry, Institute of Psychiatry (1997).

[r19] Manuel, J., Pitama, S., Clark, M., Crowe, M., Crengle, S., Cunningham, R., Gibb, S., Petrović-van der Deen, F. S., Porter, R. J., & Lacey, C. (2023). Racism, early psychosis, and institutional contact: A qualitative study of indigenous experiences. International Journal of Social Psychiatry, 69, 2121–2127. 37665228 10.1177/00207640231195297 PMC10685688

[r20] Marshall, M., Lewis, S., Lockwood, A., Drake, R., Jones, P., & Croudace, T. (2005). Association between duration of untreated psychosis and outcome in cohorts of first-episode patients: A systematic review. Archives of General Psychiatry, 62, 975. 16143729 10.1001/archpsyc.62.9.975

[r21] Mercer, L., Evans, L. J., Turton, R., & Beck, A. (2019). Psychological therapy in secondary mental health care: Access and outcomes by ethnic group. Journal of Racial and Ethnic Health Disparities, 6, 419–426. 30430460 10.1007/s40615-018-00539-8

[r22] Morgan, C., Mallett, R., Hutchinson, G., Bagalkote, H., Morgan, K., Fearon, P., Dazzan, P., Boydell, J., McKenzie, K., Harrison, G., Murray, R., Jones, P., Craig, T., & Leff, J. (2005). Pathways to care and ethnicity. 1: Sample characteristics and compulsory admission. Report from the AESOP study. The British Journal of Psychiatry, 186, 281–289. 15802683 10.1192/bjp.186.4.281

[r23] Morrison, A. P., Law, H., Carter, L., Sellers, R., Emsley, R., Pyle, M., French, P., Shiers, D., Yung, A. R., Murphy, E. K., Holden, N., Steele, A., Bowe, S. E., Palmier-Claus, J., Brooks, V., Byrne, R., Davies, L., & Haddad, P. M. (2018). Antipsychotic drugs versus cognitive behavioural therapy versus a combination of both in people with psychosis: A randomised controlled pilot and feasibility study. Lancet Psychiatry, 5, 411–423. 29605187 10.1016/S2215-0366(18)30096-8 PMC6048761

[r24] Morrison, A. P., Pyle, M., Maughan, D., Johns, L., Freeman, D., Broome, M. R., Husain, N., Fowler, D., Hudson, J., MacLennan, G., Norrie, J., Shiers, D., Hollis, C., & James, A. (2020). Antipsychotic medication versus psychological intervention versus a combination of both in adolescents with first-episode psychosis (MAPS): A multicentre, three-arm, randomised controlled pilot and feasibility study. Lancet Psychiatry, 7, 788–800. 32649925 10.1016/S2215-0366(20)30248-0 PMC7606914

[r25] Murden, R., Allan, S. M., Hodgekins, J., & Oduola, S. (2024). The effectiveness of public health interventions, initiatives, and campaigns designed to improve pathways to care for individuals with psychotic disorders: A systematic review. Schizophrenia Research, 266, 165–179. 38412687 10.1016/j.schres.2024.02.032

[r26] NHS England (2016). Implementing the early intervention in psychosis access and waiting time standard: Guidance. NHS England, London (2016). Available at https://www.england.nhs.uk/mental-health/resources/accesswaiting-time/

[r27] NHS England. (2023). NHS long term workforce plan. Department of Health and Social Care.

[r28] NICE. (2015). Psychosis and schizophrenia in adults. National Institute for Clinical Excellence.

[r29] Oduola, S., Craig, T. K. J., Das-Munshi, J., Bourque, F., Gayer-Anderson, C., & Morgan, C. (2019). Compulsory admission at first presentation to services for psychosis: Does ethnicity still matter? Findings from two population-based studies of first episode psychosis. Social Psychiatry and Psychiatric Epidemiology, 54, 871–881. 30895353 10.1007/s00127-019-01685-y PMC6656788

[r30] Oduola, S., Craig, T. K. J., Iacoponi, E., Macdonald, A., & Morgan, C. (2023). Sociodemographic and clinical predictors of delay to and length of stay with early intervention for psychosis service: Findings from the CRIS-FEP study. Social Psychiatry and Psychiatric Epidemiology, 59, 25–36. 37353580 10.1007/s00127-023-02522-z PMC10799823

[r31] Oduola, S., Craig, T. K. J., & Morgan, C. (2021a). Ethnic variations in duration of untreated psychosis: Report from the CRIS-FEP study. Social Psychiatry and Psychiatric Epidemiology, 56, 931–941. 32681277 10.1007/s00127-020-01922-9 PMC8192380

[r32] Oduola, S., Das-Munshi, J., Bourque, F., Gayer-Anderson, C., Tsang, J., Murray, R. M., Craig, T. K. J., & Morgan, C. (2021b). Change in incidence rates for psychosis in different ethnic groups in South London: Findings from the clinical record interactive search-first episode psychosis (CRIS-FEP) study. Psychological Medicine, 51, 300–309. 31739818 10.1017/S0033291719003234 PMC7893508

[r33] Oluwoye, O., Stiles, B., Monroe-DeVita, M., Chwastiak, L., McClellan, J. M., Dyck, D., Cabassa, L. J., & McDonell, M. G. (2018). Racial-ethnic disparities in first-episode psychosis treatment outcomes from the RAISE-ETP study. Psychiatric Services, 69, 1138–1145. 30152275 10.1176/appi.ps.201800067 PMC6395511

[r34] ONS (2011). *Official Labour Market Statistics.* Office for National Statistics. https://www.nomisweb.co.uk/reports/lmp/la/1946157253/report.aspx?c1=2013265927&c2=1946157256.

[r35] Pacchiarotti, I., Tiihonen, J., Kotzalidis, G. D., Verdolini, N., Murru, A., Goikolea, J. M., Valentí, M., Aedo, A., & Vieta, E. (2019). Long-acting injectable antipsychotics (LAIs) for maintenance treatment of bipolar and schizoaffective disorders: A systematic review. European Neuropsychopharmacology, 29, 457–470. 30770235 10.1016/j.euroneuro.2019.02.003

[r36] Patel, M. X., Matonhodze, J., Baig, M. K., Gilleen, J., Boydell, J., Holloway, F., Taylor, D., Szmukler, G., Lambert, T., & David, A. S. (2011). Increased use of antipsychotic long-acting injections with community treatment orders. Therapeutic Advances in Psychopharmacology, 1, 37–45. 23983926 10.1177/2045125311407960 PMC3736900

[r37] Patel, R., Oduola, S., Callard, F., Wykes, T., Broadbent, M., Stewart, R., Craig, T. K., & McGuire, P. (2017). What proportion of patients with psychosis is willing to take part in research? A mental health electronic case register analysis. BMJ Open, 7, e013113. 10.1136/bmjopen-2016-013113 PMC5353309 28279995

[r38] Perera, G., Broadbent, M., Callard, F., Chang, C. K., Downs, J., Dutta, R., Fernandes, A., Hayes, R. D., Henderson, M., Jackson, R., Jewell, A., Kadra, G., Little, R., Pritchard, M., Shetty, H., Tulloch, A., & Stewart, R. (2016). Cohort profile of the South London and Maudsley NHS Foundation Trust biomedical research Centre (SLaM BRC) case register: Current status and recent enhancement of an electronic mental health record-derived data resource. BMJ Open, 6, e008721. 10.1136/bmjopen-2015-008721 PMC4785292 26932138

[r39] Royal College of Psychiatrists (2022). National Clinical Audit of Psychosis – National report for England Early Intervention in Psychosis Audit 2021/2022. Available at https://www.rcpsych.ac.uk/improving-care/ccqi/national-clinical-audits/national-clinical-auditof-psychosis.

[r40] Schlief, M., Rich, N., Rains, L. S., Baldwin, H., Rojas-Garcia, A., Nyikavaranda, P., Persaud, K., Dare, C., French, P., Lloyd-Evans, B., Crawford, M., Smith, J., Kirkbride, J. B., & Johnson, S. (2023). Ethnic differences in receipt of psychological interventions in early intervention in psychosis services in England – a cross-sectional study. Psychiatry Research, 330, 115529. 37926056 10.1016/j.psychres.2023.115529

[r41] Singh, S. P. (2007). Outcome measures in early psychosis; relevance of duration of untreated psychosis. The British Journal of Psychiatry. Supplement, 50, s58–s63. 18019046 10.1192/bjp.191.50.s58

[r42] Singh, S. P. (2010). Early intervention in psychosis. The British Journal of Psychiatry, 196(5), 343. 20435956 10.1192/bjp.bp.109.075804

[r43] SQL – ANSI. (2011). American National Standards Institute SQL (standard|reference|specification) – SQL (92|99|2003|2011). American National Standards Institute.

[r44] StataCorp. (2017). Stata statistical software: Release 15. College Station, TX: StataCorp LLC.

[r45] Stewart, R., Soremekun, M., Perera, G., Broadbent, M., Callard, F., Denis, M., Hotopf, M., Thornicroft, G., & Lovestone, S. (2009). The South London and Maudsley NHS Foundation Trust biomedical research Centre (SLAM BRC) case register: Development and descriptive data. BMC Psychiatry, 9, 51. 19674459 10.1186/1471-244X-9-51 PMC2736946

[r46] Turpin, G., & Coleman, G. (2010). Clinical psychology and diversity: Progress and continuing challenges. Psychology Learning & Teaching, 9, 17–27.

[r47] WHO. (1996). Personal and psychiatric history schedule. Geneva: World Health Organisation.

[r48] Williams, J. C., Harowitz, J., Glover, J., Tek, C., & Srihari, V. (2020). Systematic review of racial disparities in clozapine prescribing. Schizophrenia Research, 224, 11–18. 33183948 10.1016/j.schres.2020.07.023

[r49] Wood, N., & Patel, N. (2017). On addressing ‘whiteness’ during clinical psychology training. South Africa Journal of Psychology, 47, 280–291.

