# Supplementary material for: Ethnic disparities, clinical and pathways to care characteristics associated with the offer, uptake, and type of psychological therapy during first-episode psychosis: examining the role of early intervention for psychosis
Source: Psychol Med. 2025 Sep 5;55:e262. doi: 10.1017/S0033291725101529 (PMC13040589; doi:10.1017/S0033291725101529)
Supplement: Oduola et al. supplementary material [file S0033291725101529sup001.docx]

**Title:** Ethnic disparities, Clinical and Pathways to Care characteristics associated with the Offer, Uptake, and Type of Psychological Therapy during First Episode Psychosis: Examining the Role of Early Intervention for Psychosis

**Supplementary Table S1:** Comparing ethnicity in Lambeth and Southwark (study catchment areas) with England *(from ONS 2011 Census data).*

|  | Lambeth | Southwark | England |
| --- | --- | --- | --- |
| **Ethnicity** |  |  |  |
| White | 55% | 51.4% | 81.0% |
| Mixed | 8.1% | 7.2% | 3.0% |
| Asian | 7.3% | 9.9% | 9.6% |
| Black | 24% | 25.1% | 4.2% |
| Other | 5.7% | 6.3% | 2.2% |
